# Elevated endothelin-1 levels as risk factor for an impaired ocular blood flow measured by OCT-A in glaucoma

**DOI:** 10.1038/s41598-022-15401-5

**Published:** 2022-07-12

**Authors:** Claudia Lommatzsch, Kai Rothaus, Lasse Schopmeyer, Maria Feldmann, Dirk Bauer, Swaantje Grisanti, Carsten Heinz, Maren Kasper

**Affiliations:** 1 Department of Ophthalmology and Ophtha Lab at St. Franziskus Hospital, Hohenzollernring 74, 48145 Muenster, Germany; 2 Department of Ophthalmology, University of Luebeck, Luebeck, Germany; 3 Amsterdam UMC, Location AMC, Amsterdam, The Netherlands; 4 Department of Ophthalmology, Braunschweig Hospital, Braunschweig, Germany; 5 Department of Ophthalmology, University of Essen, Essen, Germany

**Keywords:** Glaucoma, Predictive markers, Risk factors, Three-dimensional imaging

## Abstract

The purpose of this study was to ascertain whether a correlation exists between glaucoma-associated alteration of ocular vascular haemodynamics and endothelin-1 (ET-1) levels exist. Eyes of patients with cataract (n = 30) or glaucoma (n = 68) were examined with optical coherence tomography (OCT) and OCT-angiography (OCT-A; AngioVue™-RTVue-XR; Optovue, Fremont, California, USA). The peripapillary and the macular vessel density (VD) values were measured. Inferior and superior retinal nerve fibre layer (RNFL) thickness loss was used for further OCT staging. Aqueous humour of the examined eye and plasma were sampled during cataract or glaucoma surgery and analysed by means of ELISA to determine their ET-1 level. Glaucoma eyes are characterised by reductions in RNFL thickness and VD that correlate significantly with the OCT GSS score. Peripheral and ocular ET-1 level were significantly elevated in patients with glaucoma and correlate positively with the OCT-GSS score of the entire study population. Peripapillary and macula VD of glaucoma patients correlates negatively with plasma ET-1 levels. Multivariable analysis showed a subordinate role of intraocular pressure predictive factor for impaired retinal blood flow compared with plasma ET-1 level in glaucoma. Peripheral ET-1 level serves as risk factor for detection of ocular blood flow changes in the optic nerve head region of glaucomatous eyes.

## Introduction

Glaucoma occurs in all age groups, and its prevalence increases with age^1^. In view of the current demographic trends, the disease will increasingly occupy us socio-economically in the future and cause an increasing economic burden on society. The course of the disease is gradual over years and may remain asymptomatic for a very long time. Often undetected, it leads to irreversible visual field loss with blindness in the final stage^2^. The early diagnosis, and the associated therapeutic possibility of slowing down the progression of the disease is therefore of substantial importance^3^.

Glaucoma is commonly characterized by elevated intraocular pressure (IOP) associated with retinal ganglion cell (RGC) loss, eventually leading to blindness^2^. However, due to IOP lowering therapeutic treatment, IOP is not a suitable marker for monitoring glaucoma progression. In normal tension glaucoma (NTG), IOP is neither a reliable marker for diagnosis nor for glaucoma progression. Thus, open-angle glaucoma is increasingly not viewed as an isolated ocular disease that leads to progressive optic neuropathy only due to elevated IOP. Rather, there is increasing evidence that many cases of glaucoma are related to systemic diseases leading to disturbed ocular blood flow, not necessarily related to elevated IOP^4^. With regard to the possible causes of glaucoma, in addition to altered intracranial pressure^5^ and altered scleral rigidity^6^, altered systemic or ocular blood flow (OBF)^7^ must also be considered a pathogenic factor. In this vascular theory, glaucoma is seen as a consequence of insufficient ocular blood supply. This can result from either increased IOP or reduced OBF. Patients with glaucoma showed reduced OBF in the ophthalmic artery, central retinal artery and posterior ciliary arteries^8^. The perfusion of the optic disc is determined by the ocular perfusion pressure (OPP). This is the calculated difference between mean arterial blood pressure (MAP) and IOP and has been linked to glaucoma in many studies^9–11^. In addition to elevated IOP, low systemic blood pressure stands in direct relation to low OBF and low OPP, as mean ocular perfusion pressure (MOPP) is 2/3 the mean arterial pressure (MAP) subtracted by the IOP, which may result in impaired perfusion with subsequent ischaemia of the optic nerve head (ONH)^12,13^.

As the ophthalmic artery is modulated by the retinal l-arginine/nitric oxide pathway and by endothelium-derived vasoactive substances, alterations in OBF may be related to an endothelial dysfunction^14^.

In 1985 it was shown that the endothelium of blood vessels secretes a previously unknown vasoconstricting substance which was given the name of endothelium-derived constricting factor^15^. This substance was later isolated and sequenced from cultured endothelial cells and was named endothelin. Endothelin-1 (ET-1) is the most potent vasoconstrictor known. In the eye, ET-1 is synthesized by the vascular endothelium^16^, the retinal epithelium^17^ and the ciliary epithelium^18^. Various studies showed an association of elevated plasma ET-1 level with progressive glaucoma^19,20^, but another study could not verify these findings^21^. To date, very few studies have addressed the possible correlation between elevated ET-1 levels and impaired OBF by means of colour doppler imaging (CDI)^22–24^. CDI is inherently limited in its resolution of retinal blood flow velocity^25^ and is thus increasingly being replaced by the optical coherence tomography angiography (OCT-A), the most recently developed imaging technique for quantification of ocular blood flow^26^. OCT-A is a non-invasive technique that can visualise blood flow (vessel density, VD) in the different retinal layers^27,28^. We and others have shown via OCT-A analysis that the papillary and macular VD is significantly decreased in glaucomatous eyes^29–31^.

To our knowledge, no study has yet investigated the relationship of ET-1 to retinal VD by means of OCT-A imaging in glaucoma patients. Therefore, we set out to determine the concentration of ET-1 in plasma and aqueous humour (AqH) in relation to papillary and macular VD in glaucoma eyes and controls.

## Materials and methods

### Study design

This prospective monocentric study was conducted at the Department of Ophthalmology and Ophtha-Lab at St. Franziskus Hospital Muenster (Germany) and approved by the ethics committee of the Medical Association of Westfalen-Lippe, Germany (reference number 2018-331-f-S). The study adhered to the tenets of the Declaration of Helsinki. All patients provided written informed consent before study entry.

### Subjects

A group of patients with glaucoma was compared with a control group without glaucoma. In both groups there was a medical indication for cataract surgery or, in the glaucoma group, for glaucoma surgery.

The diagnosis of glaucoma was in all cases based on the findings of funduscopy and OCT. At least two of the following optic nerve criteria had to be fulfilled for inclusion: increased vertical cup-to-disc ratio (VCDR; ≥ 0.5); VCDR asymmetry > 0.2; glaucomatous reduction in peripapillary retinal nerve fibre layer (RNFL) thickness and/or ganglion cell complex (GCC) thickness at posterior pole.

Control subjects had to have IOP ≤ 21 mmHg without a history of elevated IOP or a positive family history of glaucoma. Clinically, the optic disc had to be normal in appearance with an intact neuroretinal rim, unremarkable RNFL and GCC analysis, and no lateral asymmetry in VCDR exceeding 0.1 as measured by OCT.

As lens opacities might have a significant influence on the image quality of retinal blood flow measured via OCT-A, we excluded patients with severe cataract from the control, but also from the glaucoma group. Furthermore, the exclusion criteria for both groups were any ocular disease apart from mild to moderate cataract in the glaucoma group or refractive error >  ± 6dpt sphere and ± 2dpt cylinder. Low imaging quality with OCT-A signal strength index (SSI) index < 45 and/or scan quality (SQ) < 6 resulted in exclusion. All subjects had to be at least 18 years old and have undergone no previous surgical treatment apart from uncomplicated cataract surgery with posterior chamber lens implantation or selective laser trabeculoplasty/argon laser trabeculoplasty or laser iridotomy (more than 6 months before inclusion). The presence or history of systemic vascular disease (e.g. diabetes mellitus, arterial hypertension, status post apoplexy/myocardial infarction/thrombosis) or a history of systemic vasoactive medication also led to exclusion in both groups.

### Examinations

Prior to OCT and OCT-A measurement, all patients underwent a detailed ophthalmic examination including best corrected visual acuity (BCVA), slit-lamp biomicroscopy with indirect ophthalmoscopy and measurement of mean arterial pressure (MAP). After OCT and OCT-A imaging, Goldmann applanation tonometry was performed. The relevant medical history and the current glaucoma medication were documented.

RNFL and GCC thickness, focal and global loss volume (FLV/GLV), CDR and rim volume were measured with OCT (SD-OCT, RTVue-XR; Optovue, Inc., Fremont, California, USA—software version 2016.2.035). The pupils were not dilated with medication for the measurements.

### Optical coherence tomography angiography

OCT-A (AngioVue™—RTVue-XR; Optovue, Fremont, California, USA, software version 2016.2.035) was then performed in both groups.

The OCT-A system in this study uses a split-spectrum amplitude decorrelation angiography algorithm and operates at 70,000 A-scans per second to acquire OCT-A volumes consisting of 400 × 400 A-scans. All scans taken had a 6 × 6 mm scan area centred on the fovea, and an additional 4.5 × 4.5 mm grating centred on the optic disc was used. The reduction of projection artefacts in the deep layers was automated using 3D Projection Artifacts Removal (3D PAR). In addition, all OCT-A images were reviewed by the same person (CL) to ensure correct centring and segmentation and to identify poor-quality scans with motion artefacts or blurring. Poor-quality images were excluded based on the presence of one or more of the following criteria: SSI < 45; SQ < 6; poor fixation resulting in duplication of artefacts; media opacity obscuring the view of the vasculature (e.g. vitreous opacities).

The optic disc scan was performed in the segmentation area of the superficial nerve fibre layer, referred to by the manufacturer as "radial peripapillary capillaries" (RPC), which corresponds to an extension from the internal limiting membrane (ILM) to the posterior border of the RNFL. With regard to ONH area, the software calculates the value first as whole VD (ONH Whole in %) and then divides it into an intrapapillary VD (ONH Inside in %) value and an average peripapillary VD value (PeriONH Average in %) with further subdivision into eight different peripapillary sectors (PeriONH NasSup, NasInf, InfNas, InfTemp, TempInf, TempSup, SupTemp, SupNas in %).

In the macular region segmentation was performed into superficial (SVP) and deep vascular plexus (DVP), predefined by the manufacturer as follows: SVP: ILM to inner plexiform layer (IPL) − 10 µm; DVP: IPL − 10 µm to outer plexiform layer (OPL) + 10 µm. AngioVue™ first evaluates the whole VD (Macula SVP/DVP Whole in %) and then distinguishes a foveal region (Fovea SVP/DVP in %; diameter of 1 mm) from a parafoveal region (ParaFovea SVP/DVP Average in %; diameter of 3 mm), and a perifoveal region (PeriFovea SVP/DVP Average in %; diameter of 6 mm). Finally, the system subdivides the parafoveal and perifoveal zones each into four equal quadrants (ParaFovea SVP/DVP Temp, Sup, Nas and Inf and PeriFovea SVP/DVP Temp, Sup, Nas, and Inf, in %).

The foveal avascular zone (FAZ; in mm^2^) was calculated as a combination of the two plexuses and is referred to by the manufacturer as the "retinal" segmentation layer (ILM to OPL + 10 µm). Another way of looking at the avascular zone in more detail is to describe its acircularity. AngioVue™ automatically calculates a so-called acircularity index (AI), which describes the relationship between the measured circumference of the FAZ and the circumference of the same size of a round surface. A value of 1.0 means a perfect circle. The less round the shape, the larger the calculated index.

### Glaucoma staging

The glaucoma group was classified according to disease severity using a staging tool kindly provided by Brusini et al.^32^. This tool utilizes the average peripapillary RNFL thickness in the superior and inferior region and applies age correction. Using a non-linear classification scheme, these OCT GSS (glaucoma staging system) scores were interval scaled and stratified into seven OCT GSS stages: healthy, borderline, stage 1, stage 2, stage 3, stage 4 and stage 5. Additionally, the defect type was characterised as inferior, superior or diffuse, depending on the area(s) presenting damage.

### Peripheral blood and AqH samples

Peripheral blood and AqH samples were collected from patients undergoing either cataract or glaucoma surgery (e.g. trabeculectomy, canaloplasty) who were scheduled for surgery independent of the study.

AqH was collected during the planned glaucoma or cataract surgery (100 to 150 µl), transferred to 1.5-ml reaction tubes and stored at − 80 °C until use. Venous blood was collected during the anaesthetic period (heparin blood 7 ml). The blood was centrifuged at 4 °C for 10 min at 2000×*g*, and the plasma phase was transferred to 1.5-ml reaction tubes and stored at − 80 °C until further analysis.

### Protein analysis

AqH and plasma samples were analysed for their protein content using a commercially available Bradford protein assay (Bio-Rad, Munich, Germany).

### Quantification of endothelin-1

AqH and plasma samples were analysed using the Endothelin-1 Quantikine ELISA Kit (R&D Systems, Minneapolis, USA). Standards and samples were measured in duplicate according to the manufacturer's instructions.

### Statistical analysis

MedCalc Version 12.4 (Ostend, Belgium) and R Version 4.0.2 (2020-06-22; Dormagen, Germany) were used for all statistical analyses. Normal distribution of the data was checked by means of the Shapiro test and data were expressed as mean ± standard deviation for N > 30 or gaussian distributed (parametric) observations. Otherwise, the median and the interquartile interval (25% and 75%) are given (non-parametric). We set the significance level at α = 5%. The means of two groups were compared using an adequate T-test if the preconditions were met. As non-parametric alternative we used a Wilcoxon test (rank test, or Rank sum test). If the preconditions were fulfilled, more than two groups were compared by means of an ANOVA followed by a Tukey post-hoc analysis. Alternatively, a Kruskal‒Wallis test (Dunn as post hoc) was performed. For the post-hoc analyses we carried out Bonferroni corrections of the p-values. The chi-square test was chosen for the comparison of nominal scaled observations if any matrix element was at most 5, otherwise the exact Fisher test was used.

Linear correlation analysis was performed using the Pearson test (parametric) or the Spearman rank test (non-parametric). The resulting correlation coefficient r is a measure of the effect strength. In order to determine the extent of the connection, we followed the scheme of Cohen^33^: relationships under rho (r) = 0.10 are considered insignificant, those from r = 0.30 correspond to an average effect, and those from r = 0.50 correspond to a strong effect.

For univariable regression analysis we applied Pearson’s correlation test for bivariate gaussian distributions, otherwise Spearman’s method was chosen. For multivariable regression we assumed a gaussian linear model. The resulting p-values of the independent variable allowed us to identify variables with significant influence in the multivariable regression model.

## Results

### Patients

A total of 98 eyes were included in the study (Table 1, Supplemental Table S1), 68 of which were glaucomatous eyes and 30 non-glaucomatous control eyes. The groups did not differ significantly regarding age, sex or MAP. The glaucoma group was characterized by significantly more pseudophakic lenses (p = 0.0291), higher IOP (p < 0.001), and a better visual acuity (p < 0.001) than the control group (Table 1). As a reduced visual acuity in the control group as well as the glaucoma group might be caused by the cataract associated lens opacity^34^, higher LogMar was not an exclusion criterion for these subjects. Furthermore, LogMar did not show any meaningful correlation to the OCT/OCT-A parameter (data not shown). The IOP did not differ between phakic and pseudophakic eyes (p = 0.98). Furthermore, OCT and OCT-A-analysis showed a significantly reduced GCC, RNFL, rim area, and ONH VD as well as macular VD in the superficial region of glaucoma patients compared with the controls (p < 0.001; Table 1), with no significant difference in the FAZ or Fovea SVP/DVP. In addition, FLV, GLV, vertical CDR and cup volume were significantly enhanced compared with the controls (p < 0.001; Table 1). Further clinical and OCT/OCT-A parameters are presented in Supplementary Table 1.

**Table 1:** Clinical data and OCT/OCT-A parameters.

	Control	Glaucoma	P-Value
**Clinical data**			
Eye (n)	OD:19|OS:11	OD:30|OS:38	0.13^1^
Glaucoma entity (n)	POAG:0|XFG:0|NTG:0|PG:0|ACG:0	POAG:49|XFG:9|NTG:5|PG:2|ACG:3	n.a
1st diagnos. of glaucoma (Mo)		72.00 [29.75–120.00]	n.a
Age at OP	66.77 ± 9.01	64.51 ± 12.18	0.37^2^
Sex (n)	Female:20|Male:10	Female:41|Male:27	0.71^1^
IOP (mmHg)	14.00 [12.00–14.00]	18.00 [15.00–21.25]	< 0.001^3^
Refractive error (dpt)	0.07 ± 2.88	0.00 [-1.38–0.50]	0.29^3^
Visual acuity (LogMAR)	0.40 [0.30–0.60]	0.12 [0.00–0.30]	< 0.001^3^
Previous surgeries (n)		Cataract:10 Laser trabeculoplasty:4 Laser iridotomy:3	n.a
MAP (mmHg)	95.00 [92.50–99.50]	93.33 [91.67–101.67]	0.76^3^
Topical antiglaucomatosa		3.00 [2.00–4.00]	n.a
Beta n (%)		43 (74.1%)	n.a
CAI n (%)		47 (81.0%)	n.a
PGA n (%)		51 (87.9%)	n.a
Alpha n (%)		23 (39.7%)	n.a
Pilo n (%)		2 (3.4%)	n.a
Systemic CAI n (%)		5 (8.6%)	n.a
**OCT and OCT-A parameter**			
Ganglion cell complex (µm)	96.55 ± 8.41	76.00 [67.00–84.00]	< 0.001^3^
Focal loss volume (%)	0.41 [0.21–0.80]	5.67 [2.42–9.59]	< 0.001^3^
Global loss volume (%)	2.10 [0.50–4.25]	19.24 ± 10.91	< 0.001^3^
RNFL thickness (µm)	96.79 ± 8.72	72.72 ± 13.10	< 0.001^2^
Cup/disc ratio total	0.31 ± 0.15	0.68 [0.58–0.77]	< 0.001^3^
Rim area (mm^2^)	1.37 ± 0.35	0.68 [0.49–0.91]	< 0.001^3^
Disc area (mm^2^)	2.03 ± 0.32	2.05 ± 0.34	0.78^2^
VD ONH whole	47.00 ± 2.52	35.40 [30.17–38.90]	< 0.001^3^
VD macula SVP whole	41.90 ± 4.15	37.02 ± 4.28	< 0.001^2^
VD fovea SVP	19.99 ± 6.69	17.30 [11.12–21.65]	0.06^3^
VD macula DVP whole	39.96 ± 4.37	41.90 ± 4.83	0.06^2^
VD fovea DVP	34.28 ± 8.49	32.23 ± 7.99	0.26^2^
FAZ	0.26 ± 0.10	0.26 [0.22–0.38]	0.29^3^

*n.a.* not applicable, *POAG* primary open-angle glaucoma, *XFG* exfoliative glaucoma, *NTG* normal-tension glaucoma, *PG* pigmentary glaucoma, *ACG* angle-closure glaucoma, *MAP* mean arterial pressure, *Beta* beta-blocker, *CAI* carbonic anhydrase inhibitor, *PGA* prostaglandin analogue, *Alpha* alpha-adrenergic agonist, *Pilo* pilocarpine, *RNFL* retinal nerve fiber layer, *ONH* optic nerve head, *VD* vessel density, *FAZ* foveal avascular zone, *SVP* superficial vascular plexus, *DVP* deep vascular plexus.

*p < 0.05.

^1^ Chi-square-test.

^2^t-test.

^3^Wilcoxon rank sum test.

### OCT staging tool

It was our intention to analyse the suitability of an OCT staging system for diagnostic application. Age and the superior/inferior region RNFL thickness values were applied to the OCT staging tool of Brusini et al.^32^. An OCT GSS score was computed for all eyes, and patients were classified according to the presence of RNFL defects (OCT GSS stage: healthy, borderline, stages 1 to 5), as well as defect localization (none, inferior, superior, diffuse; Supplemental Table 2).

Of the 30 control patients, seven were classified as borderline (n = 5), stage 1 (n = 1), or stage 2 (n = 1). Eleven patients of the glaucoma group were classified as healthy (Supplemental Table S2). The defect location type differed significantly among OCT GSS stages (p < 0.001). In particular, the diffuse type predominated with increasing OCT GSS stage.

**Figure 1:**
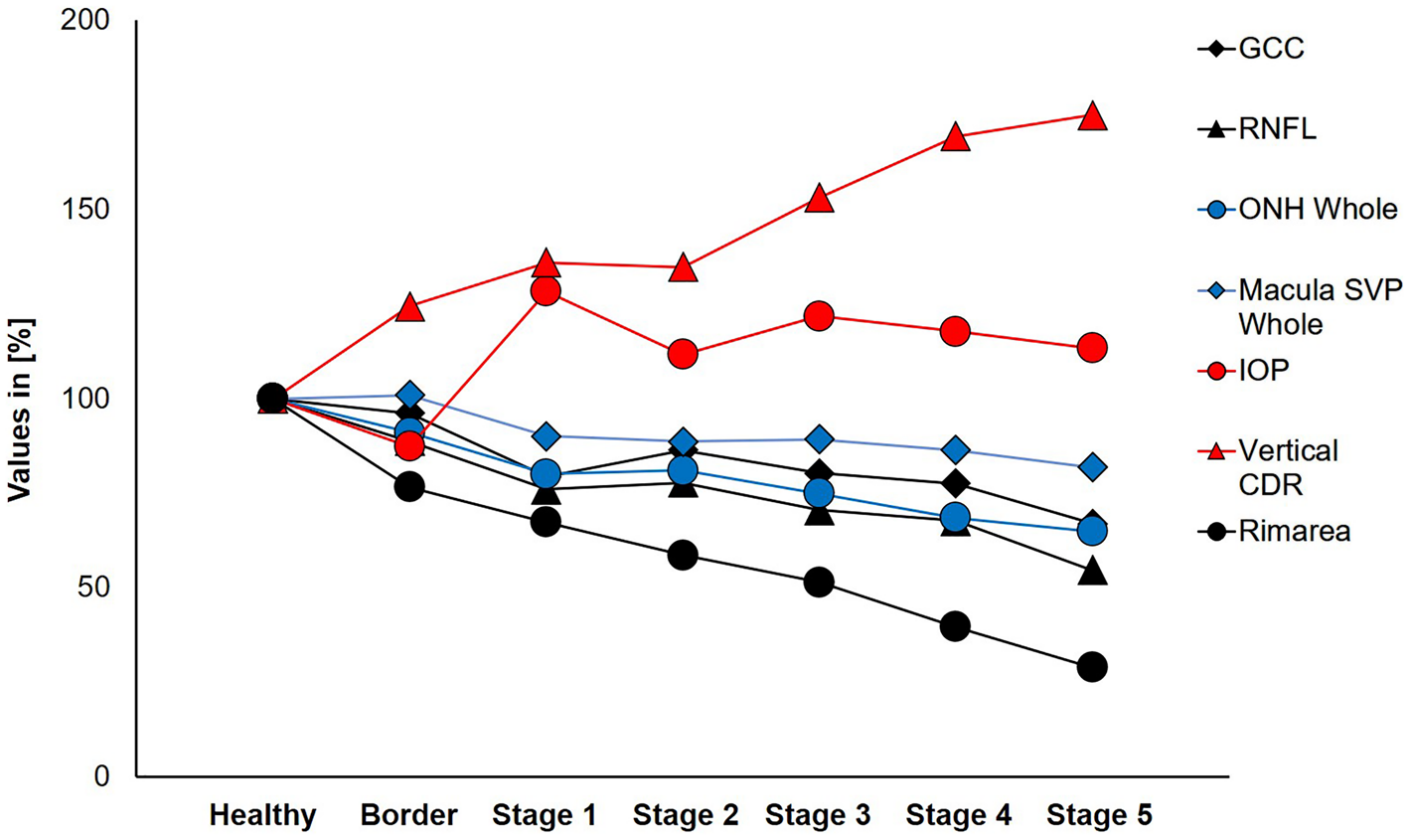
OCT and OCT-A parameter vs. OCT staging. Values from GCC, RNFL in [µm], IOP in [mmHg], ONH Whole in [%], Macula SVP Whole in [%], vertical CDR and rim area in [mm^2^] for the healthy stage were set to 100%. Values for the other stages (borderline, stage 1–5) were normalized to the healthy stage and are displayed in [%].

However, in the glaucoma group, all different OCT stages (p = 0.43), and their defect locations (p = 0.61) were commonly distributed within all glaucoma entities. The correlation of the OCT and OCT-A parameters with the OCT GSS score showed in particular that the GCC (r = − 0.723; p < 0.001), the RNFL (r = − 0.912; p < 0.001), the rim area (r = − 0.764; p < 0.001), ONH Whole (r = − 0.771; p < 0.001) and the VD of the whole-macula SVP (r = − 0.545; p < 0.001) decreased significantly with increasing OCT GSS score (Fig. 1). In contrast, vertical CDR (Fig. 1; r = 0.775, p < 0.001), cup volume (r = 0.486, p < 0.001) and IOP (Fig. 1; r = 0.379, p < 0.001) correlated significantly positively with OCT GSS score (Fig. 1). Furthermore, the visual acuity (LogMar) correlated significantly negatively (r = − 0.370; p < 0.001) with the OCT GSS score. RNFL thickness was significantly lower in the borderline stage than in the healthy stage (adj. p-value < 0.01; Supplemental Table 2).

### Increased level of endothelin-1 in glaucoma

**Figure 2:**
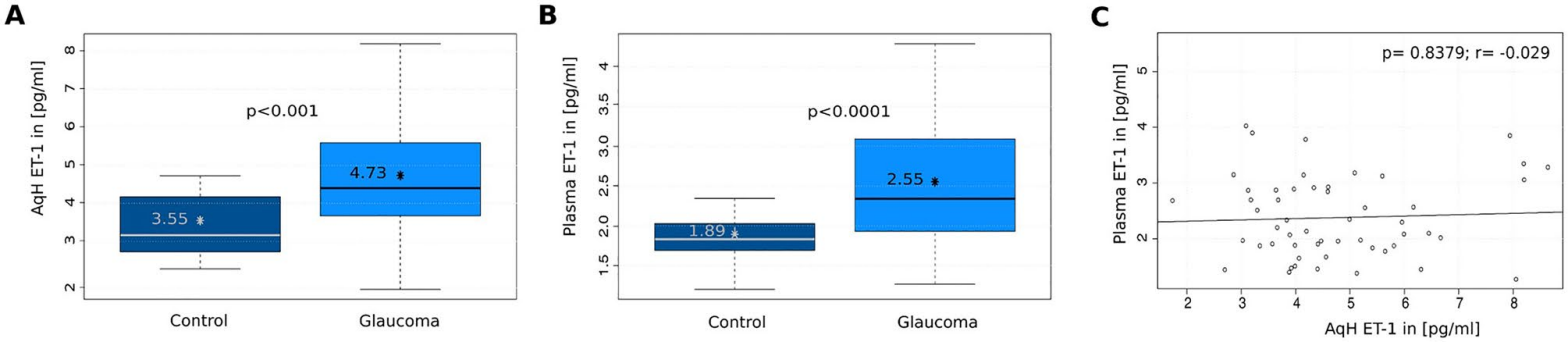
(**A**,**B**) ET-1 level of (**A**) AqH and (**B**) plasma samples from cataract and glaucoma patients. (**C**) Spearman rank correlation of plasma ET-1 level to AqH ET-1 level in the glaucoma patients.

Quantification of ET-1 levels in AqH and plasma samples showed significant elevation of ET-1 level in glaucoma patients compared with the control group in both tissues (AqH p < 0.001; plasma p < 0.001 (Fig. 2A,B; Supplementary Table 1).

The ET-1 level in the AqH of glaucomatous eyes was significantly higher than in the respective plasma samples (p < 0.001), but did not correlate with the plasma ET-1 level (p = 0.84; r = − 0.029; Fig. 2C). Similar to the glaucoma group, ET-1 level in AqH did not correlate with the ET-1 plasma level in the control group. However, univariable regression analysis including samples from both control and glaucoma groups showed no significant correlation between AqH and plasma ET-1 level (p = 0.4335; rho = 0.092).

Correlation analysis for AqH and plasma levels of ET-1 in the glaucoma group (Table 2; more detail in Supplemental Table 3) showed that elevated AqH ET-1 level did not correlate with the IOP or lens status, but correlated significantly positively with the number of topically applied antiglaucoma medications (p < 0.01, r = 0.374; Supplemental Table 3). To ensure that the positive correlation between the concentration of ET-1 in the AqH and topical antiglaucomatosa was not due to the fact that the study participants with a higher IOP used more pressure-lowering eye drops, we performed a subgroup analysis by IOP. When the glaucoma groups were subdivided by IOP (Group 1 IOP ≤ 18 mmHg Group 2 IOP 19–24 mmHg Group 3 IOP ≥ 25), there was no significant difference in the number of topical antiglaucomatosa between the three IOP groups (Kruskal Wallis p = 0.4938; data not shown). Furthermore, elevated plasma ET-1 level of glaucoma patients correlated significantly positively with age (p = 0.035; Table 2), refraction (p = 0.031; Table 2), vertical CDR (p = 0.044; Table 2) and disc area (p < 0.01; Table 2) and significantly negatively with IOP (p = 0.030; Table 2). Additionally, the plasma ET-1 level of patients with glaucoma correlated significantly with reduced superior RNFL thickness (p = 0.049 Table 2), while no correlation was found with reduced inferior RNFL thickness (Table 2), nor between AqH ET-1 level and superior/inferior RNFL thickness loss (Table 2, Supplemental Table 3).

**Table 2:** Correlation of ET-1 level in plasma or AqH to OCT/OCT-A parameters in glaucoma.

Parameter	ET1-Plasma [pg/ml]		ET1-AqH [pg/ml]	
	rho	(p)	rho	(p)
Age at surgery	0.256	(0.035)	− 0.178	(0.20)
Refractive error	0.264	(0.031)	− 0.088	(0.53)
Visual acuity (LogMAR)	0.131	(0.29)	− 0.060	(0.67)
IOP	− 0.263	(0.030)	− 0.020	(0.89)
Cup/disc ratio vertical	0.261	(0.044)	0.029	(0.85)
Disc area	0.370	(0.004)	0.024	(0.87)
ONH Whole	− 0.080	(0.52)	0.066	(0.64)
PeriONH TempInf	− 0.246	(0.05)	0.201	(0.17)
PeriONH TempSup	− 0.348	(0.007)	− 0.001	(1.00)
Fovea SVP	− 0.323	(0.007)	0.177	(0.20)
Fovea DVP	− 0.331	(0.006)	0.204	(0.14)
FAZ	0.256	(0.035)	− 0.110	(0.43)
Topical antiglaucoma medications	− 0.126	(0.31)	0.374	(0.006)
RNFL Thickness Sup	− 0.242	(0.050)*	0.101	(0.48)
RNFL Thickness Inf	− 0.089	(0.48)	0.086	(0.55)
OCT GSS score	0.112	(0.38)	− 0.042	(0.78)

The p-value p = 0.050* in the table indicates the significance of the corresponding test, that means p < 0.05.

^a^Correlation coefficient: Pearson’s rho for bivariate gaussian distributions, otherwise Spearman’s rho.

^b^Correlation test: Pearson’s method for bivariate gaussian distributions, otherwise Spearman’s method.

Furthermore, several negative correlations between plasma ET-1 level and VD were identified (Table 2, Supplemental Table 3): In the peripapillary region, there was a negative correlation with the plasma ET-1 level in the temporal superior sector (PeriONH TempSup). In the macular region, the measurement areas Fovea SVP, Fovea DVP and the FAZ correlated negatively with the plasma ET-1 level (Table 2; Supplemental Table 3). There was no significant correlation between AqH and ET-1 levels in the glaucoma group.

ET-1 levels in both plasma (r = 0.296, p < 0.01) and AqH (r = 0.269, p = 0.024) correlated significantly positively with the OCT GSS score of the entire study population. To determine whether the elevated ET-1 level might be related to an enhanced protein concentration in the respective samples from glaucoma patients, we analysed the protein concentration of AqH and plasma samples. Protein concentration was found to be significantly elevated compared with the control group (Supplementary Table 1) and correlated positively with the elevated ET-1 level measured in the respective plasma samples (AqH r = 0.22, p = 0.13; plasma r = 0.33, p < 0.01). Furthermore, the plasma protein concentration correlated positively with increasing OCT GSS score (r = 0.23; p = 0.031), but no further correlations with age, IOP, lens status or OCT parameters were detected.

### Multivariable analysis: plasma ET-1 level as predictor of retinal VD

In order to identify correlation and dependency structures among the variables, we conducted a multivariable analysis. We used plasma ET-1 level, MAD, IOP and age as independent variables and analysed whether they were suitable markers for the modulated parameters of glaucomatous eye measured via OCT-A.

At first, we assessed the predictive value of those markers in healthy and glaucoma patients (Supplemental Tables 4, 5). The greatest number of significant differences (hereinafter: most significances) was found between IOP and OCT-A parameters (e.g. ONH Whole, Macula SVP Whole), pointing to IOP being an important predictor for the presence of glaucoma, while AqH and plasma ET-1 level were of less importance.

In particular, plasma ET-1 level showed the most significances (e.g. ONH Whole, PeriONH TempInf and TemSup in Fovea SVP and Fovea DVP), while the changes in the FAZ were not predicted by plasma ET-1 level (Supplemental Table 4). AqH ET-1 level (Supplemental Table 5), age and MAD (Supplemental Tables 4, 5) showed fewer significances for prediction of OCT-A parameters.

When focussing the multivariable analysis on the glaucoma group only, IOP, age, plasma, and AqH ET-1 levels were used as independent variables (Supplemental Tables 6, 7). Analysis showed no significances for IOP to predict OCT-A parameters. Plasma ET-1 level was most significant for predicting the PeriONH TempSup, Fovea SVP, and Fovea DVP. The FAZ remained unsignificant (Supplemental Table 6). No predicting factors for AqH ET-1 level were found (Supplemental Table 7).

### Differentiation between normal (normal) and elevated (high) plasma ET-1 level in glaucoma

In accordance with previous studies by Emre et al. and Cellini et al., we defined ranges to subclassify all patients according to their plasma ET-1 level into *normal* and *high* ET-1 groups^19,20^. The *normal* range was defined according to the mean ± SD and min/max ET-1 level of the control group (1.785 ± 0.33; min 1.75 pg/ml/max 2.5 pg/ml; Supplementary Table 8).

Within glaucoma group, we identified 37 patients with *normal* plasma ET-1 level (1.92 ± 0.33 pg/ml) and 31 patients with significantly elevated (*high*) plasma ET-1 level (3.13 [2.87–3.44] pg/ml; p < 0.001). The ET-1 level in AqH did not differ significantly between the two groups (*normal* 4.74 ± 1.21 pg/ml; *high* 4.18 [3.25–5.43]).

The patients of the *high* ET-1 group were not significantly older than those of the *normal* group (p = 0.06), had a higher protein concentration in plasma samples (p = 0.021; AqH p = 1.000), and showed a significantly reduced IOP (p = 0.045) (Supplemental Table 8).

The *normal* and *high* groups did not differ with regard to defect type, OCT staging or the main OCT-A parameters such as GCC, RNFL and VD (Supplementary Table 8).

## Discussion

The factors causing the development and progression of glaucoma are not yet fully understood. In Caucasians, hypertensive glaucoma is the most common entity and is easily detected by measurement of IOP. Elevated IOP (≥ 21 mmHg) is considered to be a risk factor and marker for diagnosis of hypertensive glaucoma^35^, but not for NTG (IOP ≤ 21 mmHg)^36^. Furthermore, many people with an IOP of ≥ 21 mmHg have no glaucoma^37^. As therapeutic treatment of hypertensive glaucoma is also focused on IOP lowering drugs, early diagnosis and clinical long-term monitoring of glaucoma based on IOP measurement is inappropriate.

Furthermore, most patients with progressive glaucoma show few or no symptoms^38^. Patients usually only notice visual impairment by peripheral and eventually central vision loss in late stages of the disease. Visual field measurement by perimetry is still considered the diagnostic gold standard today, despite the drawbacks of the test–retest variability^39^, which relies on the nature of the psychophysical examination method itself. One of the disadvantages of the resulting functional parameters is that they are based on a psychophysical examination and are therefore strongly influenced by the attention, motivation and reaction time of patients. Pathophysiologically it is assumed that about 40% of the retinal ganglion cells have already been irreversibly lost before changes in perimetry can be detected^40^. Besides IOP measurement and perimetry, glaucomatous changes of the retinal structures are diagnosed early by RNFL thickness loss as measured by OCT.

Based on changes of the superior/inferior RNFL thickness, a severity classification of ocular changes can be calculated using the OCT GSS^32^. Our study confirmed the ability of the OCT GSS of Brusini et al. to assign patients accurately to seven categories: healthy, borderline and five different stages of glaucoma severity. Even though, we did not include perimetry measurements, our results showed a significant correlation of reduced visual acuity with increased OCT GSS score. Therefore, we could confirm the suitability of the OCT staging tool for a standardized and objective classification of glaucomatous RNFL damage. However, RNFL-thinning can reach a stage where any further decrease is not detectable by OCT, called the “floor effect”^41^. There are new insights that OCT-A can monitor the disease by means of VD for later progression stages than OCT by RNFL. Additionally, it appears that OCT-A might be able to detect structural glaucomatous damage in the form of reduced VD in earlier stages than OCT is able to detect RNFL changes^42–44^.

Since it was shown that the RNFL based OCT GSS scores correlated significantly with the impaired haemodynamic parameters measured via OCT-A, inclusion of OCT-A parameters for glaucoma staging might be of added value in the future if these results can be confirmed with larger patient populations.

In order to strengthen the early diagnosis of glaucoma before symptom onset, it would be of great interest to identify IOP-independent glaucoma biomarkers (e.g. in blood or AqH), and correlate them with simpler non-invasive tests such as OCT/OCT-A.

One possible biomarker for altered retinal blood flow is endothelin, which has been implicated in glaucomatous pathogenesis^45–47^. Various experimental studies have shown a direct effect of ET-1 to increase IOP for example by modulation of the AqH outflow^48–53^. While Chortiz et al. showed a positive correlation of elevated ET-1 in AqH with elevated IOP for the entire study population, no significant correlation has been shown in the subgroup analysis of patients with cataract, POAG or exfoliation glaucoma (XFG), or with regard to the ET-1 plasma level and IOP^54^.

In contrast, when analysing the entire study population in the current study, no correlation between ET-1 AqH or ET-1 plasma with IOP was found, neither after subdivision into three IOP-ranges IOP (Low = IOP ≤ 18 mmHg, middle = 19–24 mmHg and High =  ≥ 25 mmHg; data not shown). In line with the study of Chortiz et al., our study did not show a correlation between ocular or plasma ET-1 levels and elevated IOP in subgroup analysis.

In healthy humans, Polak et al. showed that exogenous ET-1 administration resulted in significant reduction of blood flow at the ONH and in the choroid as measured with laser Doppler flowmetry^55^. Schmetterer et al., using colour Doppler ultrasound, also showed that ET-1 dose-dependently reduced fundus pulsations in the macula and the optic disc, but did not affect blood flow velocity in the ophthalmic artery or systemic haemodynamics^56^, indicating that these areas appear to be especially sensitive to ET-1.

Previous studies have detected ET-1 levels in AqH in glaucoma patients that were both significantly higher than in controls and significantly higher than in corresponding plasma samples^54,57–61^. We confirmed these findings in our current study.

With regard to the plasma ET-1 levels in healthy persons and glaucoma patients, studies have inconsistently reported unaltered^54,57,58,62,63^, or elevated ET-1 levels in the plasma of glaucoma patients^19,64^.

Our study showed significant elevation of ET-1 in both AqH and plasma samples from patients with glaucoma compared with the controls. We confirmed the positive correlation of plasma ET-1 level with patient age shown previously by Maeda et al.^65^. In line with previous studies^54,57^, no correlation could be found between intraocular and plasma ET-1 levels, implying that the ET-1 in the AqH is formed and released in the eye.

Moreover, we detected elevated protein concentrations in plasma samples, as also shown by others (Koliakos et al. 2004)^66,67^. Unlike Koliakos et al. (Koliakos et al. 2004), we found a positive correlation between the elevated protein and ET-1 level in plasma samples of glaucoma patients but no further correlation with age, IOP or OCT parameters.

In the current study, plasma ET-1 level correlated positively with the OCT GSS score^32^ and with the vertical CDR, which may indicate that the plasma ET-1 level is related to more advanced stages of glaucoma and accompanied RNFL thinning.

Previous studies distinguished between normal and elevated ET-1 level in glaucoma patients^19,20^ and related the high ET-1 level to a progression in visual field defects. In contrast, another study found no significant correlation between levels of plasma ET-1 and severity of glaucoma, as classified by visual field testing and RNFL thickness^21^. However, we could not find any significant differences in visual acuity, OCT or OCT-A parameters when comparing glaucoma patients with normal and high plasma ET-1 level.

Functional studies on the effect of ET-1 on the retinal blood flow are contradictory. Polak et al. showed that exogenous ET-1 administration in healthy humans resulted in significant reduction of blood flow at the ONH and in the choroid as measured with laser Doppler flowmetry^55^. Schmetterer et al., using colour Doppler ultrasound, also showed that ET-1 dose-dependently reduced fundus pulsations in the macula and the optic disc, but did not affect blood flow velocity in the ophthalmic artery or systemic haemodynamics^56^.

The results of the current study showed a significant correlation between elevated ET-1 plasma level in glaucoma patients and an impaired retinal blood flow at the ONH, supporting the findings of Polak et. al.^55^ and indicating that impaired blood flow in these areas might be in causal relation to the peripheral ET-1 level.

Previous studies distinguished between normal and elevated ET-1 level in glaucoma patients^19,20^ and related the high ET-1 level to a progression in visual field defects. However, we could not detect any significant differences in visual acuity, OCT or OCT-A parameter when comparing glaucoma patients subdivided in normal and high plasma ET-1 level.

Several studies assumed a correlation of elevated ocular or peripheral ET-1 level with impaired retinal blood flow, as measured by CDI, in various ocular diseases, e.g. NTG or XFG diabetic retinopathy and retinitis pigmentosa^22–24,68^.

The results of the current study are in line with those studies, showing a significant correlation between elevated ET-1 plasma level in glaucoma patients and an impaired retinal blood flow at the ONH. The OCT-A analysis in our study showed, in the glaucoma group, a significant correlation between impaired VD, in particular of the PeriONH TempSup and Fovea (SVP/DVP) and FAZ, and elevated ET-1 level in plasma samples. However, no correlation could be shown for the elevated AqH ET-1 level in glaucoma patients. This might reflect the relevance of systemic factors leading to alterations in the retinal blood flow and subsequently to glaucoma. A recent retrospective longitudinal study by Kiyota et al.^69^ showed a significant correlation between impaired ONH perfusion of the superior and temporal quadrant measured by laser speckle flowgraphy (LSFG) in the form of the tissue area mean blur rate (MT) in POAG, and their respective circumpapillary RNFL thinning. Therein it was demonstrated that the lower MT of the superior and temporal quadrant in POAG was associated with faster RNFL thinning in the respective quadrants during follow-up period of at least 2 years compared to the inferior quadrant. These ocular changes were significantly corelated with an increased age.

Kiyota et al. suggested that age is a contributing factor in particular because of the relation to cardiac dysfunctions and proposed that age related systemic circulatory failures may lead to ONH hypoperfusion observed in glaucoma.

In the current study the elevated ET-1 plasma level correlates strongly with age, as well as impaired VD of the PeriONH TempSup. The peripheral plasma ET-1 level is shown to be of superior predictive significance compared with age or IOP within the glaucoma cohort.

However, as compared with Kiyota et al., in this study patients with cardiac dysfunction and hypertension were excluded, and yet we found a strong correlation between ET-1 and impaired blood flow, which points to an early biomarker potential of plasma ET-1 levels.

It remains to be investigated whether the elevated ET-1 in glaucoma originates from the intraocular nervous damage itself or is secondary to other systemic processes that lead to elevated ET-1. The fact that this study found significant correlations with plasma ET-1 but no correlation with AqH ET-1, points to the secondary, systemic origin. As ET-1 is, next to its potent vasoconstrictive effects, known to be associated with inflammatory response, it might be of interest to examine whether the seen correlations of plasma ET-1 with RFNL thickness and VD are also associated with an altered cytokine pattern^70^.

Further investigations, in particular with longitudinal perspectives and a broader glaucoma cohort with and without further systemic diseases, are needed to support the biomarker quality of plasma ET-1 for prediction of ONH blood flow.

Multivariable regression analysis confirmed these findings, showing that factors such as age and MAP are of less importance as predictive factors for both glaucoma and impaired retinal blood flow. While the multivariable analysis confirmed that IOP is a predictive factor for glaucoma, plasma ET-1 level turned out to be a more significant predictor than IOP for impaired retinal blood flow at the ONH and in the foveal area within the glaucoma group.

Interestingly, plasma ET-1 level in the multivariable analysis serves as a predictor for impaired VD of the foveal area, while the FAZ is unaffected. Our previous studies showed that the FAZ is affected in glaucoma at later stages^71^ congruent with the fact that the patients included in the current study had less severe glaucomatous damage (only 6 of 68 eyes were in group 5 of the Brusini classification). Thus, plasma ET-1 level may be an early predictive factor of impaired VD within PeriONH TempSup and the Fovea SVP/DVP area, independent of IOP.

A previous study in healthy eyes showed a IOP independent autoregulatory mechanism for ONH blood flow^72^, pointing to a dysregulation in glaucoma patients. As ONH lacks a normal blood brain barrier^73,74^, peripheral circulating vasoconstrictive molecules as endothelin might have direct access to vascular smooth muscle cells^75,76^. Thus, a causal relation between the elevated ET-1 plasma level of glaucoma patients and the impaired blood flow in particular of the ONH region might exist.

### Limitations of the study

In this study glaucoma patients with predominantly early or mild stages of glaucoma were included. We expected perimetry to not have shown many visual field defects, whereas RNFL changes could be observed by performing OCT, and promising results regarding sensitivity and specificity of the OCT GSS have been demonstrated^32^. As perimetry remains the current gold standard in glaucoma monitoring, this technique should nevertheless be included in future studies, as well as expansion of the patient population to patients with more advanced stages of glaucoma and more individuals with NTG and XFG. The expansion and re-evaluation of the initial patient population at a later point of time would also allow to determine the sensitivity and specificity of plasma ET-1 as a diagnostic index.

The blood samples were taken while the patients were under general anaesthesia. An increase in plasma ET-1 level during surgical operations has been demonstrated^77^. However, the ET-1 level increases gradually after 2 h of surgery^78^ and declines slowly thereafter. In the current study all samples were obtained within the first 10 min after onset of anaesthesia. Thus, an influence of anaesthesia on the ET-1 level is unlikely.

In the glaucoma group, the glaucoma medication was not interrupted preoperatively. It is conceivable that individual drugs had an influence on the ET-1 concentration.

For analysing the correlation between the proteins and the clinical parameter we did no adjustment of the p-values for multiple testing. Therefore, our observations of this pilot study have to be confirmed, by tested with a larger number of cases for the sub group analysis. Furthermore, individuals with glaucomatous symptoms (e.g. elevated IOP), ocular complications, and systemic diseases should be included. In particular, the presence of an associated systemic disorder might influence the plasma ET-1 level.

## Conclusion

This study detected a notable correlation of ET-1 level with the OCT GSS score as determined using the classification of Brusini et al. and with impaired peripapillary and foveal VD on SVP and DVP as measured by OCT-A. Furthermore, multivariable regression analysis indicated that plasma ET-1 level is a significant predictive marker for impaired retinal blood flow in the ONH region in particular in glaucoma.

## Supplementary Information

Supplementary Tables.

## Data Availability

The data that support the findings of this study are available from the corresponding author upon reasonable request.

## Abbreviations

ACG: Angle-closure glaucoma
AI: Acircularity index
Alpha: Alpha-adrenergic agonist
AqH: Aqueous humour
Bet: Beta-blockers
BCVA: Best corrected visual acuity
CDR: Cup-to-disc ratio
CAI: Carbonic anhydrase inhibitors
CDI: Colour Doppler imaging
DVP: Deep vascular plexus
ELISA: Enzyme-linked immunosorbent assay
ET-1: Endothelin-1
FAZ: Foveal avascular zone
FLV: Focal loss volume
GCC: Ganglion cell complex
GLV: Global loss volume
GSS: Glaucoma staging system
ILM: Internal limiting membrane
IPL: Inner plexiform layer
IOP: Intraocular pressure
MAP: Mean arterial pressure
NTG: Normal-tension glaucoma
OBF: Ocular blood flow
OD: Oculus dexter
ONH: Optic nerve head
OPL: Outer plexiform layer
OPP: Ocular perfusion pressure
OS: Oculus sinister
OCT: Optical coherence tomography
OCT-A: Optical coherence tomography angiography
PG: Pigmentary glaucoma
PGA: Prostaglandin analogues
Pilo: Pilocarpine
POAG: Primary open-angle glaucoma
XFG: Exfoliation glaucoma
RNFL: Retinal nerve fibre layer
SQ: Scan quality
SSI: Signal strength index
SVP: Superficial vascular plexus
VCDR: Vertical cup-to-disc ratio
VD: Vessel density
